# Physical Activity for Quiescent and Mildly Active Inflammatory Bowel Disease: A Systematic Review and Meta-Analysis

**DOI:** 10.1093/jcag/gwad021

**Published:** 2023-08-10

**Authors:** Banke Oketola, Olayinka Akinrolie, Sandra Webber, Nicole Askin, Rasheda Rabbani, Ahmed M Abou-Setta, Harminder Singh

**Affiliations:** Applied Health Sciences Program, Faculty of Graduate Studies, University of Manitoba, Winnipeg, Canada; Department of Internal Medicine, Max Rady College of Medicine, Rady Faculty of Health Sciences, University of Manitoba, Winnipeg, Manitoba, Canada; Applied Health Sciences Program, Faculty of Graduate Studies, University of Manitoba, Winnipeg, Canada; Department of Physical Therapy, College of Rehabilitation Sciences, Rady Faculty of Health Sciences, University of Manitoba, Winnipeg, Canada; Winnipeg Regional Health Authority Virtual Library Department, Neil John Maclean Health Sciences Library, University of Manitoba, Winnipeg, Manitoba, Canada; Department of Internal Medicine, George & Fay Yee Center for Healthcare Innovation, Rady Faculty of Health Sciences, University of Manitoba, Winnipeg, Manitoba, Canada; Department of Community Health Sciences, Max Rady College of Medicine, Rady Faculty of Health Sciences, University of Manitoba, Winnipeg, Manitoba, Canada; Department of Internal Medicine, George & Fay Yee Center for Healthcare Innovation, Rady Faculty of Health Sciences, University of Manitoba, Winnipeg, Manitoba, Canada; Department of Community Health Sciences, Max Rady College of Medicine, Rady Faculty of Health Sciences, University of Manitoba, Winnipeg, Manitoba, Canada; Department of Physical Therapy, College of Rehabilitation Sciences, Rady Faculty of Health Sciences, University of Manitoba, Winnipeg, Canada; Department of Internal Medicine, Max Rady College of Medicine, Rady Faculty of Health Sciences, University of Manitoba, Winnipeg, Manitoba, Canada

**Keywords:** inflammatory bowel disease (IBD), physical activity, quiescent IBD, exercise, systematic reviews, inconsistent measurement

## Abstract

**Background:**

Physical activity (PA) may benefit people with inflammatory bowel diseases (IBD) by improving immunological response, musculoskeletal function, and psychological health.

**Aims:**

We distilled available evidence on the efficacy and safety of PA to improve health-related quality of life (HRQoL) and relieve persistent symptoms of fatigue, joint pain, abdominal pain, stress, anxiety, and depression in individuals with quiescent/mild IBD.

**Methods:**

We searched for trials in eight databases and trial registries. Trials using PA as an adjunct therapy in the management of adults (≥18 years) with quiescent or mild IBD, published in English between 2011 and 2023 were identified. Summary effect estimates were expressed as standardized mean difference (SMD) or mean difference (MD) with 95% confidence interval (CI) using random-effects model.

**Results:**

From the 10,862 citations retrieved, we included seven randomized controlled trials (RCTs) and one non-RCT. There was no evidence of benefit of PA on HRQoL (SMD 0.34, 95%CI −0.08 to 0.77; I^2^ 57%); high heterogeneity was noted among included trials. PA was found to be efficacious in reducing anxiety (SMD −0.35, 95%CI −0.65 to −0.05; I^2^ 0%). There was insufficient evidence to make conclusions regarding changes in fatigue, joint pain, abdominal pain, stress, and depression. All trials deemed physical activity safe.

**Conclusions:**

PA contributes to reducing anxiety in quiescent/mild IBD. There is marked heterogeneity in methodology among trials investigating PA in adults with quiescent/mild IBD. This review highlights the need for consistent definitions of PA types and intensities in this field of research.

## Introduction

Inflammatory bowel disease (IBD) is a chronic inflammatory condition of the gastrointestinal tract comprising two main forms: Crohn’s disease (CD) and ulcerative colitis (UC).^1^ IBD is a lifelong condition with no cure and can be associated with substantial long-term morbidity.^2,3^ Intestinal manifestations of IBD include abdominal pain, diarrhea, malabsorption of nutrients, and gastrointestinal blood loss.^4,5^ Extraintestinal manifestations, which affect 25% of patients^6^ include fatigue, joint pain, erythema nodosum, iritis and uveitis, low bone mineral density, and loss of muscle mass.^7,8^ Although the symptom burden is worse during periods of active mucosal inflammation, many symptoms persist after individuals achieve remission.^4^

While most of the focus has been on the management of the active disease phase in IBD, with recent major advances in management of active IBD, burden of symptoms in quiescent disease needs more attention.^9^ Medical therapy for management of quiescent IBD targets prevention of active inflammation and aims to delay/inhibit relapses.^10^ However, several studies have shown that individuals with quiescent IBD report physical symptoms including fatigue, abdominal pain, and joint pain; and psychological symptoms including stress, anxiety, and depression.^7,11–17^ These persisting symptoms impact health-related quality of life (HRQoL),^1,9,16^ and have cost implications for health care. Consequently, individuals with IBD continue to seek adjunct therapies.^10,18^

Previous research suggests that PA could be an adjunct therapy to help reduce ongoing symptoms in quiescent IBD and improve HRQoL.^1,8,10,18,19^ The World Health Organization (WHO) defines PA as “any bodily movement produced by skeletal muscles that requires energy expenditure…including movement during leisure time, for transport to and from places, or as part of a person’s work”.^19^ Exercise, as defined by the WHO, is a “subcategory of physical activity that is planned, structured, repetitive, and purposeful in the sense that the improvement or maintenance of one or more components of physical fitness is the objective”.^19^ In this article, we use PA as an encompassing term to refer to all forms of musculoskeletal activities including exercise.

PA may counteract some IBD-specific complications by improving immunological response and psychological health, and by reversing the decrease in muscle mass and strength.^11^ However, there is a lack of estimate on magnitude of PA’s efficacy, its acceptability and safety among individuals with IBD. This may contribute to why a large proportion of individuals with IBD do not participate in PA at recommended levels.^20^

We performed a systematic review and meta-analysis to distil the evidence on the efficacy of PA to reduce symptoms in quiescent/mild IBD. Our specific objectives were:

1. To identify, critically appraise, and summarize available evidence on the efficacy of PA as an adjunct therapy to improve HRQoL and reduce symptoms of fatigue, joint pain, abdominal pain, stress, anxiety, and depression among individuals with quiescent or mildly active IBD.
2. To assess the safety of, and adherence to, PA as an adjunct therapy in the management of quiescent or mildly active IBD.

## Methods

We followed review guidelines detailed in the Cochrane Handbook for Systematic Reviews of Interventions^21^ and reported according to the Preferred Reporting Items for Systematic Reviews and Meta-Analyses (PRISMA).^22^ The International Prospective Register of Systematic Reviews (PROSPERO) registration ID is CRD42022298310.

### Criteria for selection of studies for review—Inclusion and exclusion criteria

We included randomized controlled trials (RCTs) and nonrandomized/noncontrolled trials (non-RCTs) involving adults with quiescent or mild IBD, where any form of PA was used as an intervention in conjunction with usual care (medical therapy or no therapy). While RCTs provide the highest level of evidence, we decided to include non-RCTs to capture additional evidence of effectiveness. Our primary outcome was HRQoL. Secondary outcomes were fatigue, abdominal pain, joint pain, stress, anxiety, depression, safety of PA, and adherence. Only studies published in English, between 2011 and 2023 were included.

### Search strategy

The search strategy was developed initially in MEDLINE (Ovid) by an information specialist (NA) and peer-reviewed by a second librarian using the PRESS (Peer Review of Electronic Search Strategies) checklist.^23^ The final search strategy was translated and run in EMBASE (Ovid), Cochrane CENTRAL (Ovid), PsycInfo (Ovid), CINAHL (EBSCO), Scopus (Elsevier), Web of Science Core (Clarivate), and PEDro (web) (Supplementary Data). We searched for ongoing studies by reviewing trial registries (e.g., ClinicalTrials.gov). The most recent search was performed on January 10, 2023.

We performed a hand search of the reference lists of eligible citations and conference proceedings (2016 to January 2023) of Digestive Diseases Week, Canadian Digestive Diseases Week, World Physiotherapy Congress, and Canadian Physiotherapy Association Congress. Lastly, we screened the first 10 pages of results in Google Scholar using the search string exercise|“physical activity” ibd|“inflammatory bowel”|crohns|colitis. All citations obtained from the literature search were exported to Endnote (version 20, Clarivate) for reference management and deduplication. Relevant citations were imported into Rayyan^24^ for study selection.

### Study selection

Identification of eligible citations was carried out independently by two reviewers (BO and OA/SW) and was conducted in two stages—title/abstract screening and full-text screening. Prior to commencement of citation screening, both reviewers pilot tested the eligibility criteria on ten randomly selected titles to ensure the eligibility criteria were clear and consistently applied. Studies including other interventions in addition to PA were excluded (except when the intervention was given to all study groups equally). All conflicts that arose were resolved by discussion among the primary reviewers. When there was no resolution after discussion, a third reviewer (AMAS and/or HS) was consulted.

### Data processing and management

A Microsoft Excel data extraction form developed using the DECiMAL guide^25^ was used for data extraction. Two reviewers (BO and OA) independently carried out data extraction. The data extraction form was pilot tested on two of the eligible studies to ensure the form captured all essential components needed to satisfy the research objective. The form was used to collect author/year, demographic information of participants, number of participants, type of intervention used, primary and secondary outcomes, as well as the outcome measures used. Disagreements were resolved by discussion.

### Risk of bias assessment and methodological quality

Risk of bias (RoB) of included RCTs was assessed using the Cochrane Risk of Bias tool (version 1) for RCTs.^21^ The study quality of the non-RCT was assessed using the Newcastle-Ottawa scale.^26^ All assessments were conducted independently by two reviewers (BO and OA). Conflicts were resolved by discussion or consultation with a third reviewer (AMAS).

### Data analysis

We analyzed data collected from included trials using Review Manager (RevMan) version 5.4. Summary effect estimates were expressed as standardized mean difference (SMD) (when studies used different outcome measures) or mean difference (MD) (when studies used the same outcome measures) with 95% confidence interval (CI) using random-effects model. The sample size and means/SD for three-armed studies were combined prior to analysis. Heterogeneity was quantified using the I^2^ statistic. Source of heterogeneity was explored when I^2^ was found to be greater than 50%, after confirming accuracy of the extracted data.^21^ Due to the limited data identified, we were unable to perform subgroup analysis by sex, IBD diagnosis (CD or UC), and intensity of PA (mild, moderate, or vigorous), which was planned a-priori. We retrieved missing data from tables, figures, Supplementary Data, and we contacted authors to get additional data and clarification. Publication bias could not be assessed due to the small number of included trials.

### Quality of evidence

We assessed quality of the evidence using the Grading of Recommendations, Assessment, Development and Evaluation (GRADE) for each outcome.^27^ We rated the evidence as “high”, “moderate”, “low”, or “very low” certainty in accordance with this approach.

## Results

**Figure 1.**
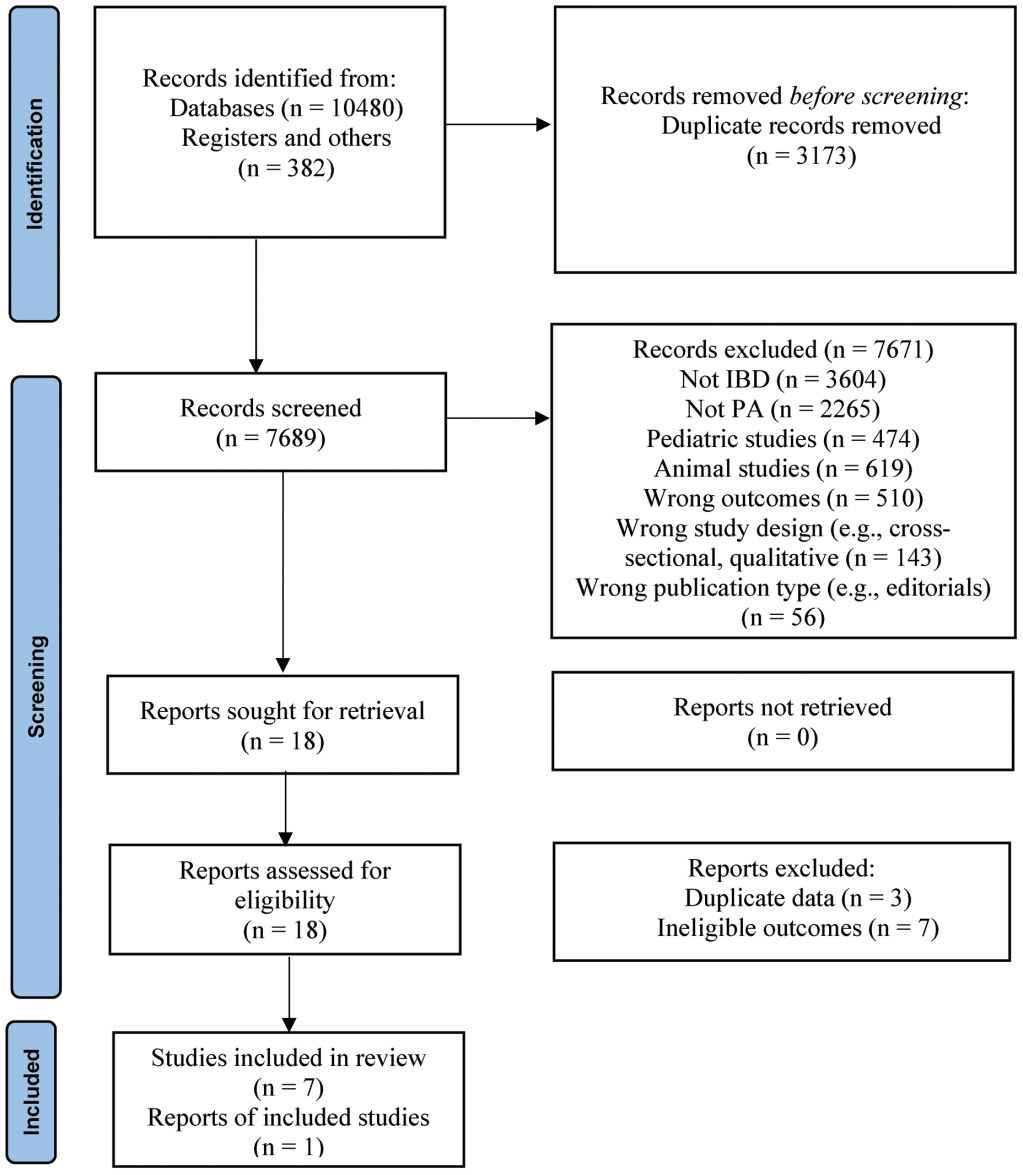
Summary of literature review and screening process—PRISMA flow diagram.

From 10,862 retrieved citations, we included seven RCTs^28–34^ and one non-RCT^5^ that met the inclusion criteria (Figure 1). Descriptive information of included studies is presented in Table 1.

**Table 1.** Characteristics of included trials.

Author/year	Mean age ± SD	Total participants	Diagnosis. Disease severity.	Authors’ definitions of disease activity	PA intervention	Authors’ description of intensity	Control group	Intervention duration	Duration Frequency of PA
Cramer et al., 2017^32^	45.5 ± 11.9	77	UC Quiesc.	Rachmilewitz clinical activity index ≤ 4	Yoga (Exercise class =1/week Home = daily).	Not defined	Medical Therapy	12 weeks	45 min Daily
Cronin et al., 2019^34^	25 ± 6.5	14	IBD Quiesc.	‘Low scores’ Simple Colitis index = 1.5 (1, 2.75) Harvey Bradshaw Index = 1 (0, 1.5)	Aerobic and resistance training (with machines/free weights)	Mod intensity defined as 5-7 out of 10 on Borg RPE	Usual care	8 weeks	Not reported 3 days/week
Jones et al., 2020^30^	49.3 ± 13.0	47	CD Quiesc.+	Calprotectin <250 μg/g CDAI < 150 CDAI = 150–219	Resistance training (squats, lunges, rope-skipping using body weight and Theraband for resistance)	Mod- to high-intensity self-rated with Resistance Intensity Scale for Exercise	Medical Therapy	26 weeks	60 mins 3 days/week
Klare et al., 2015^28^	41.1 ± 14.1	30	IBD Quiesc.+	CDAI <220 Rachmilewitz Index < 11 points	Running program (Outdoors)	Mod intensity defined as being able to talk while running	Medical Therapy/ No therapy	10 weeks	Not reported 3 days/week
Seegar et al., 2020^31^	Not reported	45	CD Quiesc.+	CDAI < 220	Grp 1-Resistance training (push ups, sit-ups). Grp 2- Aerobic Ex (jogging, cycling)	Mod intensity rated with Borg RPE and set at 60-80% of HRmax.	Medical Therapy	12 weeks	30–40 mins 3 days/week
Sharma et al., 2015^33^	Not reported	100	IBD Quiesc.	Truelove and Witts = Mild CDAI < 150	Yoga	Not defined	Medical Therapy	8 weeks	60 mins Daily
Tew et al., 2019^29^	36.9 ± 11.2	36	CD. Quiesc.+	Calprotectin <250 μg/g CDAI < 150 CDAI = 150 - 219	Leg cycle ergometer: Grp 1-HIIT= 1 min × 90% + 1 min × 15% Wpeak for 20 minutes. Grp 2-MICT= 30 min × 35% Wpeak.	Peak Power Output (Wpeak) - determined for each individual at baseline.	Medical Therapy	12 weeks	~30 mins 3 days/week
Van Erp et al., 2021^5^	45 ± 2.6	25	IBD Quiesc.	Calprotectin < 100	30 mins aerobic ex (cycling, treadmill) + 30 min resistance training with machines	Intensity for aerobic training set at 65-80% of HRmax.	Medical Therapy	12 weeks	60 mins 3 days/week

*Note:* Quiesc, Quiescent; Quiesc+, Quiescent + Mildly active; Grp 1, Group 1; Grp 2, Group 2.

Tew et al.^29^ used high-intensity interval training (HIIT) and moderate-intensity continuous training (MICT) as PA interventions. Authors measured intensity using “peak power output” (Wpeak) with 90% Wpeak for HIIT and 35% Wpeak for MICT. Jones et al.^30^ utilized resistance training as their PA intervention. They had a target intensity of “moderate to hard” for resistance training intervention which was assessed by the Resistance Intensity Scale for Exercise outcome measure. Klare et al.^28^ utilized moderate intensity for their running intervention which was defined as being able to talk while running. Seegar et al.^31^ also utilized moderate intensity which was defined as 60%–80% of maximum heart rate. They had one group performing resistance training and the second group performing aerobic exercises. The authors^32,33^ who used yoga as PA intervention did not mention intensity. Due to the variability in the types of PA and in the definitions used to describe intensity of PA in these trials, we were not able to assess the type and intensity of PA that is more beneficial for individuals with IBD.

### Risk of bias assessment and methodological quality

**Figure 2.**
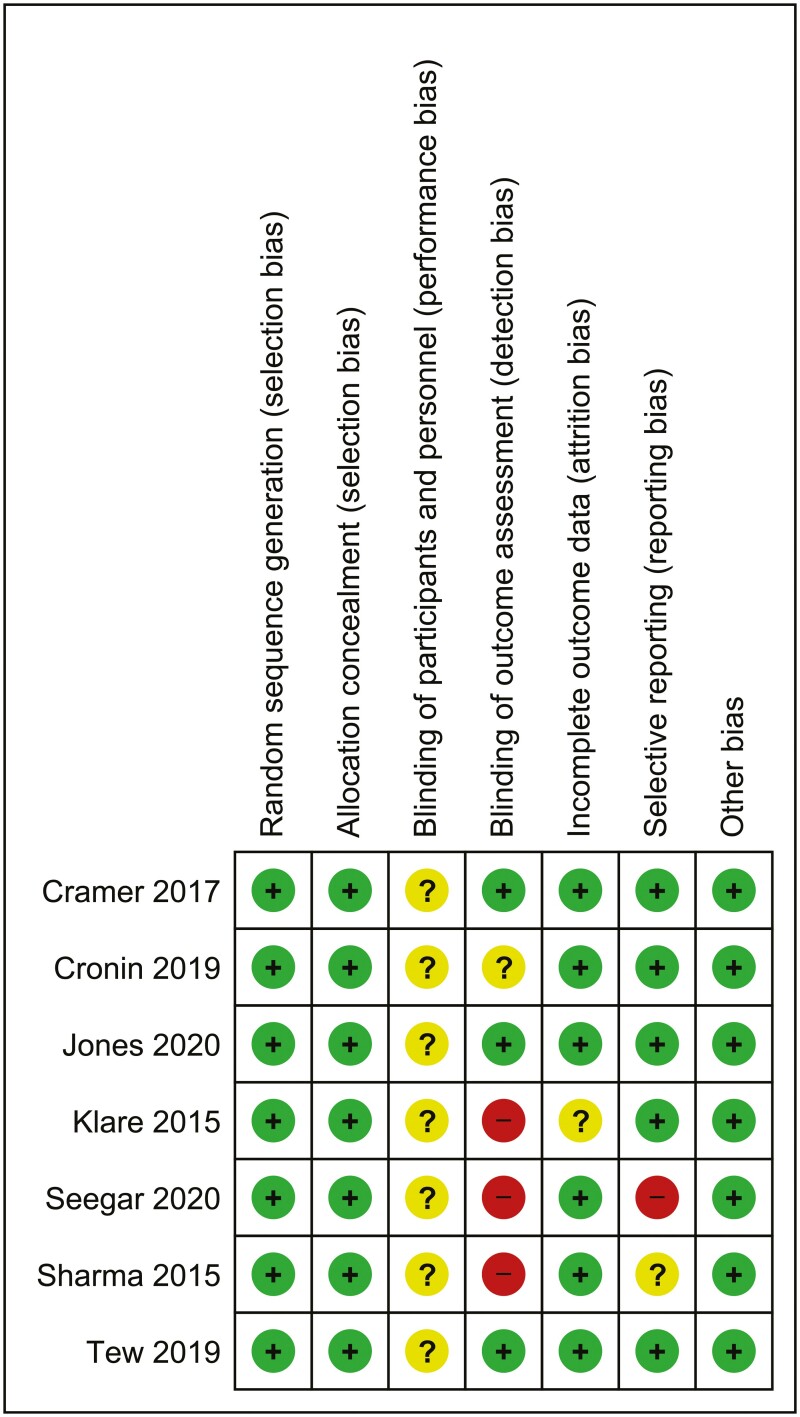
Risk of bias summary.

RoB summary of RCTs is presented in Figure 2. All RCTs were rated as having an overall unclear to high risk of bias. Masking of participants to treatment allocation was not possible in all the trials due to the nature of the intervention. In most trials, participants were their own outcome assessors; however, three trials^29,30,32^ reported masking of assessors. Masking of outcome assessment was not reported in one trial.^28^ Loss to follow-up was minimal in most trials except one^31^ with moderate loss to follow-up, suggesting attrition bias. Published protocols were not available for four of the trials,^31–34^ thus there is possibility of bias due to selective reporting.

The included non-RCT^5^ scored 5 stars out of a possible 9 stars. It was given a score of 3 stars for selection, no star for comparability and two stars for outcome, with possibility of bias resulting from lack of controlling for covariates.

### Primary outcome

**Figure 3.**
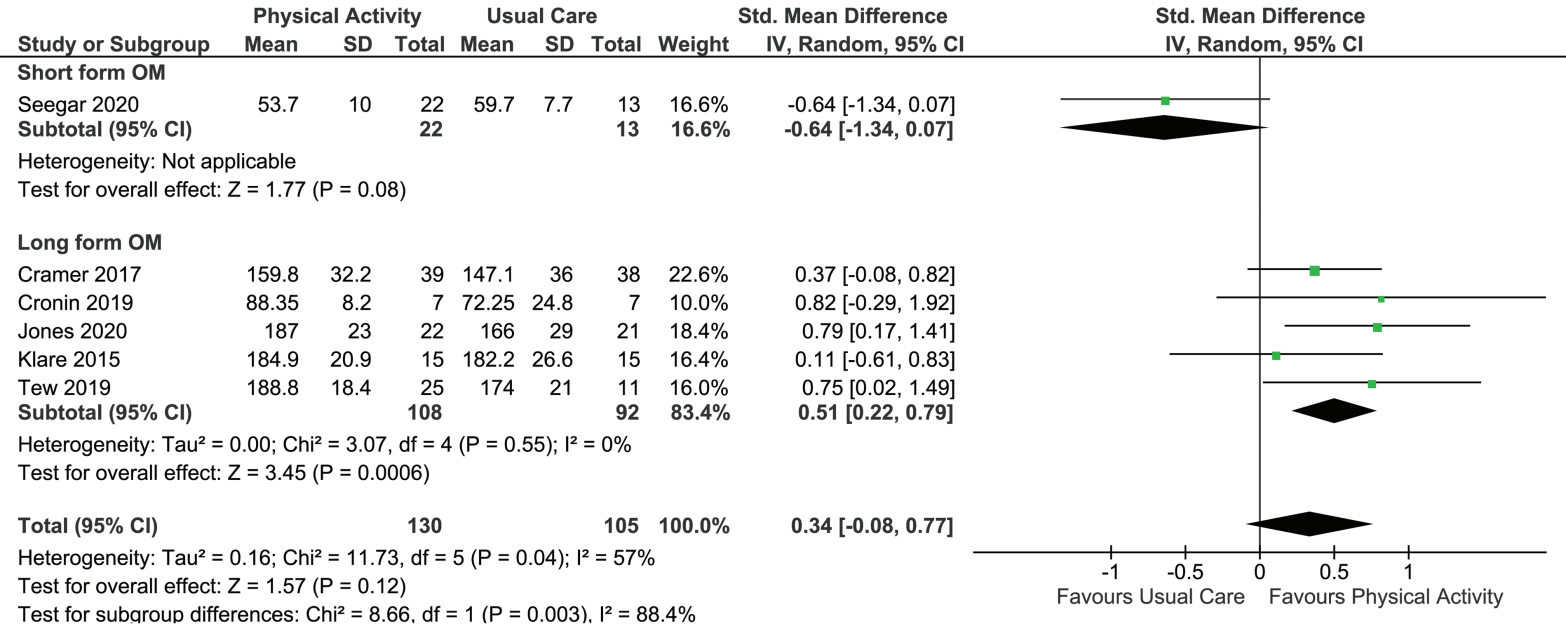
Sub-group analysis on effect of PA on HRQoL (short vs. long form outcome measures).

Our initial analysis revealed there was no evidence of benefit of PA on HRQoL (SMD 0.34, 95% CI −0.08 to 0.77; I^2^ 57%, six RCTs, 235 participants; very low certainty evidence). Due to the substantially high I^2^, heterogeneity was explored. The trial by Seegar et al.^31^ was observed to be an outlier with an opposite effect compared to the other trials in the analysis. They used the short-form of the Inflammatory Bowel Disease Questionnaire (IBDQ) while other trials used the full IBDQ and SF-36. We performed a subgroup analysis based on outcome measures (short vs. long versions). This analysis revealed a significant difference in the effect of PA on HRQoL in studies that used the full versions of the outcome measures (SMD 0.51, 95% CI 0.22 to 0.79; I^2^ 0%, five RCTs, 200 participants; moderate certainty evidence; Figure 3).

The non-RCT included in this article^5^ investigated the effect of an aerobic and resistance training program delivered for 60 min, 3 times/week for 12 weeks on HRQoL. Using the IBDQ, they reported a statistically significant improvement in HRQoL from 156 ± 21 to 176 ± 19, with change score of 20 ± 4.47.

### Secondary outcomes

#### Fatigue

Two trials^29,30^ evaluated the effect of PA on fatigue and both used the IBD Fatigue Scale as outcome measure. High heterogeneity was found between the two studies (I^2^ = 87%, Figure 5, Supplementary Data), thus meta-analysis was not performed.

Tew et al.^29^ in their pilot trial assessed the effect of HIIT and MICT over 12 weeks. After 12 weeks of HIIT, fatigue increased from 8.2 ± 3.0 to 8.3 ± 3.2 but reduced to 7.5 ± 2.5 after another 12 weeks of follow-up. In the MICT group, fatigue increased from 7.8 ± 5.3 to 8.3 ± 4.9 after 12 weeks of intervention, but also reduced to 7.3 ± 4.2 after another 12 weeks of follow-up. In the control group, fatigue scores reduced from 9.3 ± 4.1 at baseline to 7.8 ± 4.2 at 12 weeks and to 7.5 ± 4.0 after another 12 weeks of follow-up.

Jones et al.^30^ assessed the effect of moderate-high intensity resistance training. They reported a statistically significant reduction in fatigue from 6 ± 4 to 5 ± 3 after 24 weeks of intervention (*P* = 0.03), while fatigue in the control group increased from 9 ± 4 to 10 ± 5.

The non-RCT^5^ reported that following a 12-week intervention of aerobic and resistance training, fatigue in individuals with quiescent IBD reduced from 49 ± 4.7 to 29 ± 8.9 (MD −20, 95% CI: −16 to −24).

#### Anxiety

**Figure 4.**
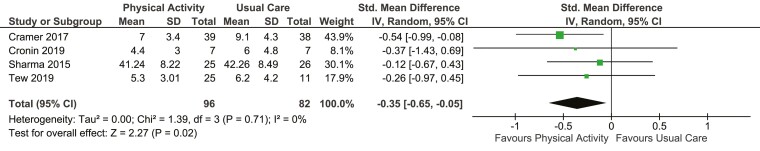
Effect of PA on anxiety.

There was a significant decrease in anxiety with PA (SMD −0.35, 95% CI −0.65 to −0.05; I^2^ 0%, four RCTs, 178 participants: low certainty evidence; Figure 4).

#### Depression

The meta-analysis of this outcome identified moderately high heterogeneity (I^2^ 54%, three RCTs, 127 participants). All studies used the Hospital Anxiety and Depression scale (HADS) as an outcome measure. No individual factors could be identified to delineate the three studies to justify a subgroup analysis.

Tew et al.^29^ reported a reduction in depression scores from 3.6 ± 3.1 to 2.7 ± 1.7 following 12 weeks of intervention in the HIIT group, and from 3.8 ± 2.9 to 2.7 ± 3.3 in the MICT group. Cronin et al.^34^ found an increase in depression scores from 1.71 ± 2.8 to 2.28 ± 3.3 after 8 weeks of combined aerobic and resistance training, and a change from 4.71 ± 4.6 to 4.14 ± 4.5 in the control group. Cramer et al.^32^ reported a statistically significant reduction in depression from 6.1 ± 3.9 at baseline to 4.5 ± 2.9 after 12 weeks of yoga in the intervention group; and 6.5 ± 3.0 to 6.9 ± 4.0 in the control group, with a group difference of 2.3 (1.0; 3.7) at 95% CI.

#### Joint pain, stress and abdominal pain

One of the included trials^33^ investigated the effect of PA on joint pain. Ten out of twenty-five participants reported joint pain at baseline: five and three reported joint pain after 4 and 8 weeks of yoga, respectively. In the control group, five out of twenty-six participants reported joint pain at baseline, six and five reported joint pain at 4 and 8 weeks, respectively.

Cramer et al.^32^ reported a reduction in perceived stress (Perceived Stress Questionnaire) from 50.0 ± 16.7 to 42.1 ± 18.7 in the yoga group compared to a reduction from 56.9 ± 14.7 to 54.8 ± 19.0 in the control group with a group difference of 7.8 (1.0; 14.6) at 95% CI, following 24 weeks of follow-up.

None of the included studies reported on abdominal pain.

#### Safety and adherence

The safety of PA was determined by the number of participants reported to experience adverse events during the intervention period. Reported adverse events (AE) from all eight trials include complaints of dizziness, nausea, and lightheadedness (n = 9), any new diagnosis (n = 2), and increased IBD activity (n = 7). There was no evidence of more individuals having adverse effects due to PA (Risk ratio 2.07, 95% CI 0.7 to 5.49; I^2^ 0%; seven RCTs, 174 participants).

Adherence was determined by the rate of attendance at PA sessions. The reasons reported for missing PA sessions included work-related tiredness, holidays, other commitments, joint discomfort, illness, bad weather, and lack of motivation. Adherence rates ranged from 47% to 87% (average of 69% across all studies). The location of PA (gym, home or outdoors) did not seem to influence adherence rates (Table 2).

**Table 2.** Safety and adherence of physical activity in the included trials.

Author/year	No. of participants	Diagnosis	Supervision (group vs. individual training)	Intervention Duration	Loss to f/u (Intervention)	Adherence (Location)	Safety (no. of participants with reported AE)	
							PA	No-PA
Cramer et al., 2017^32^	77	UC	Supervision provided at exercise class Group + Individual	12 weeks	12	60.8% (Ex class)	3	1
Cronin et al., 2019^34^	14	IBD	Supervised (not specified)	8 weeks	1	87.5% (Gym)	1	1
Jones et al., 2020^30^	47	CD	12 out of 78 sessions were supervised. (not specified)	26 weeks	1	62% (Gym)	3	2
Klare et al., 2015^28^	30	IBD	Supervised (Not specified)	10 weeks	3	80% (Outdoors)	1	0
Seegar et al., 2020^31^	45	CD	Not supervised (Individual)	12 weeks	2	47% (Home)	2	0
Sharma et al., 2015^33^	100	IBD	Supervised 1 of 8 weeks. Unsupervised 7 of 8 weeks. (Individual)	8 weeks		78.5% (Home)	0	0
Tew et al., 2019^29^	36	IBD.	Supervised (Not specified)	12 weeks		HIIT = 62% MICT = 75% (Ex class)	4	
Van Erp et al., 2021^5^	25	IBD	Supervised (Group)	12 weeks	3	Not Reported (Gym)	1	N/A

## Discussion

This systematic review identified seven RCTs and one non-RCT with a total of 300 participants. Therefore, there is a limited amount of RCT level research evaluating PA in mild/quiescent IBD. The PA interventions evaluated include yoga, moderate- and high-intensity aerobic exercises, and resistance training. The most common duration of intervention was 12 weeks. Pooled results of trials suggested that PA may be beneficial in improving HRQoL in those with quiescent/mild IBD (moderate certainty evidence), although one trial which used a different, though validated outcome measure (sIBDQ), found the opposite effect and hence more data are required. PA was found to be efficacious in alleviating anxiety (low certainty evidence). There were inconclusive data with regards to benefit of PA on fatigue, depression, stress, joint pain, and abdominal pain. Results from studies in this review suggest PA is safe for individuals with quiescent/mildly active IBD, with average adherence rates of 69% (Table 3).

**Table 3.** GRADE assessment of the quality of evidence in included trials.

Question: PA compared to medical therapy/usual care for IBD												
Certainty assessment							No. of patients		Effect		Certainty	Importance
No. of studies	Study design	Risk of bias	Inconsistency	Indirectness	Imprecision	Other considerations	PA	Medical therapy/usual care	Relative (95% CI)	Absolute (95% CI)		
Health related quality of life (assessed with: SIBDQ, SF-36)												
6	Randomised trials	Very serious^a^	Not serious	Not serious	Serious^b^	None	130	105	—	SMD 0.34 SD higher (0.08 lower to 0.77 higher)	⨁◯◯◯ Very low	CRITICAL
Certainty of evidence was graded down due to imprecision (small sample sizes) and methodological limitations (lack of blinding of outcome assessors in most studies and possible attrition bias in one of the studies). Anxiety (assessed with: HADS)												
4	Randomised trials	Serious^c^	Not serious	Not serious	Serious^d^	None	96	82	—	SMD **0.35 SD lower** (0.65 lower to 0.05 lower)	⨁⨁◯◯ Low	
Certainty of evidence was graded down by two levels due to study limitations (two of the studies had an overall unclear risk of bias and one had an overall high risk of bias) and imprecision (two of the studies had small sample sizes) HQoL-sensitivity analysis (assessed with: SIBDQ, SF-36)												
5	Randomised trials	Serious	Not serious	Not serious	Not serious	None	108	92	—	SMD 0.51 SD higher (0.22 higher to 0.79 higher)	⨁⨁⨁◯ Moderate	

Setting: Clinical setting.

*Note:* CI, confidence interval; MD, mean difference; SMD, standardized mean difference.

^a^50% of the studies are judged to be of high risk of bias.

^b^The lower boundary of the CI is negative and crosses the line of no effect.

^c^Two of the studies are unclear and one high risk of bias.

^d^Small sample size.

^e^Some level of heterogeneity.

^f^Small sample size from the studies.

PA has been reported to be an important complementary therapy in the management of diverse chronic conditions^35–41^ and in the management of IBD.^5,11,42^ However, findings of our review demonstrate that the marked heterogeneity in current trials make it challenging to determine if there are consistent beneficial effects of PA for individuals with quiescent/mild IBD. This lack of clarity may be responsible for the poor PA participation reported among individuals with IBD.^43,44^ Available trials on this subject have employed different types of PA and intensities of PA, making the determination of effect size of the efficacy of PA difficult.

There are barriers to participating and adhering to an exercise regimen in the general population and people with IBD face additional obstacles, related to persistent symptoms, which affect their ability to take part in PA.^14,43,45^ This further emphasizes the importance of standardized definitions to ensure consistency in trials on PA in the IBD population.

To the best of our knowledge, there is no recent similar systematic review on the effect of PA on persistent symptoms in quiescent/mild IBD. Previous reviews were either not recent^46^ or included trials published prior to the recent advances in medical therapies.^47^ While narrative reviews^48^ may come to similar conclusions as systematic reviews, they lack the same level of methodological rigor or transparency.

Although we conducted a systematic review using standard methodological practice, there were notable limitations. The variability in the included studies and inability to perform subgroup analysis by sex, specific diagnosis, type of PA, and intensity represent limitations. Secondly, none of the included studies used objective measures to assess amount of PA, and participation in PA could not be objectively verified. It is also important to highlight that PA participation in these studies may be by individuals who already value the benefits of PA. This may introduce unintended selection bias to the included trials. Furthermore, since participants of included studies could not be blinded to their interventions, participants may have reported more positive outcomes on their subjective assessments. The time limit applied to eligible studies may have contributed to the limited data obtained in our review. However, this time limit is relevant to focus on PA effect in the context of recent advances in medical therapy on disease processes for IBD.

This review is relevant in that it contributes to emerging literature on the efficacy and safety of PA in the IBD population. It emphasizes the marked methodological heterogeneity in available trials and the need for more RCTs to make more definitive conclusions on the efficacy of PA as an adjunct therapy in quiescent/mild IBD.

## Conclusions

There remains limited evidence to assess the efficacy of PA to improve HRQoL and manage specific persistent symptoms in quiescent or mild IBD. Rigorous and larger RCTs are needed to provide evidence-informed recommendations to individuals living with IBD.

## Supplementary Material

gwad021_suppl_Supplementary_Data

## Data Availability

The data underlying this article are available in the article and in Supplementary Data.
