# Supplementary material for: Physical Activity for Quiescent and Mildly Active Inflammatory Bowel Disease: A Systematic Review and Meta-Analysis
Source: J Can Assoc Gastroenterol. 2023 Aug 10;6(5):162–71. doi: 10.1093/jcag/gwad021 (PMC10558194; doi:10.1093/jcag/gwad021)
Supplement: gwad021_suppl_Supplementary_Data [file gwad021_suppl_supplementary_data.docx]

**Supplementary Data**

Supplementary Figure 5: Forest Plot – Fatigue


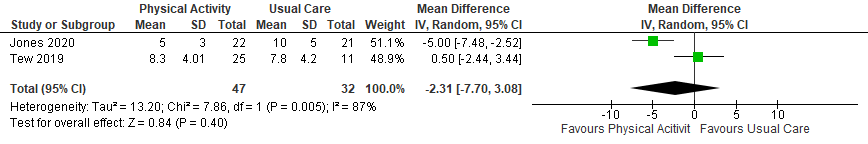


MEDLINE SEARCH STRATEGY (10^th^ January 2023)

| 1 | exp inflammatory bowel diseases/ | 86475 |
| --- | --- | --- |
| 2 | (IBD or inflammatory bowel* or crohn* or cleron disease or enteritis or enterocolitis or ileocolitis or ileitis or ileitide* or colitis or proctocolitis or pancolitis or proctosigmoiditis or rectocolitis or rectosigmoiditis or (chronic* adj2 (inflammat* or ulcer*) adj3 (colon* or bowel* or intestinal or intestine?)) or colorectitis or proctitis).ti,ab,kf | 158963 |
| 3 | or/1-2 | 168420 |
| 4 | exp exercise/ or exp exercise movement techniques/ or exp exercise therapy/ or dancing/ or gardening/ or exp sports/ | 352973 |
| 5 | (exercis* or physiotherap* or kinesiotherap* or aerobics or athletic* or workout or gym or work* out or (activity adj2 prescri*)).ti,ab,kf | 373072 |
| 6 | (physical* adj3 (activ* or fit* or condition* or program* or train*)).ti,ab,kf | 169229 |
| 7 | (activ* adj3 (motor or living or play* or gaming or game* or lifestyle or behavio?r* or transport* or commut* or transit* or travel* or aerobic*)).ti,ab,kf | 166343 |
| 8 | (crossfit* or HIIT or HIIE or plyometric* or personal train* or bodybuild* or body build* or weightlift* or (weight* adj2 lift*)).ti,ab,kf | 8066 |
| 9 | ((train* or condition* or program* or session*) adj2 (weight* or strength* or enduranc* or resistance or athletic* or fitness or aerobic* or interval* or flexibility or balance or muscle* or muscular or circuit or isometric* or isotonic* or agility or power)).ti,ab,kf | 77348 |
| 10 | (treadmill* or walk* or jog* or swim* or running or runner* or marathon* or triathl* or dance or dancing or climb* or cycling or bicycl* or skateboard* or rowing or rower or skate or skating or ski or skiing or hike or hiking or jump or jumping).ti,ab,kf | 390660 |
| 11 | (tai ji or tai chi or yoga or pilates or stretching or movement or qigong or qi gong or barre or tae bo).ti,ab,kf | 300633 |
| 12 | (gardening or stair* or housework or yardwork* or exergam* or wii fit or zwift or peloton or kinect or ergometer*).ti,ab,kf | 27353 |
| 13 | (martial art* or boxing or kickboxing or karate or judo or jiu jitsu or jiujitsu or taekwondo or tae kwon do or fencing or wrestl* or aikido or fight* or grappl*).ti,ab,kf | 44725 |
| 14 | (sport* or golf* or aquatic* or gymnastic* or tennis or badminton or volleyball or ball or basketball or baseball or bowling or lacrosse or racquet* or softball or polo or cricket or hockey or football or soccer or rugby or handball or mountaineer* or "track and field").ti,ab,kf | 198996 |
| 15 | or/4-14 | 1475873 |
| 16 | 3 and 15 | 2601 |
| 17 | (randomized controlled trial or clinical trial or clinical trial phase i or clinical trial phase ii or clinical trial phase iii or clinical trial phase iv or controlled clinical trial or pragmatic clinical trial).pt | 907112 |
| 18 | exp "clinical trials as topic"/ or exp epidemiologic studies/ | 3085975 |
| 19 | (random* or nonrandom* or pseudorandom* or placebo* or groups).ab | 3137040 |
| 20 | trial.ti,ab | 659306 |
| 21 | (case control or case series or cohort* or longitudinal or retrospective or prospective or interrupted time series or cross sectional).tw | 2291836 |
| 22 | ((Follow up or followup or control*) adj2 (study or studies)).tw. | 302962 |
| 23 | or/17-22 | 6533422 |
| 24 | 16 and 23 | 828 |
| 25 | exp animals/ not humans.sh | 4881276 |
| 26 | 24 not 25 | 767 |
| 27 | limit 26 to english language | 725 |
| 28 | limit 27 to yr="2011 -Current" | 430 |

EMBASE SEARCH STRATEGY (10^th^ January 2023)

| 1 | exp inflammatory bowel disease/ | 164216 |
| --- | --- | --- |
| 2 | (IBD or inflammatory bowel* or crohn* or cleron disease or enteritis or enterocolitis or ileocolitis or ileitis or ileitide* or colitis or proctocolitis or pancolitis or proctosigmoiditis or rectocolitis or rectosigmoiditis or (chronic* adj2 (inflammat* or ulcer*) adj3 (colon* or bowel* or intestinal or intestine?)) or colorectitis or proctitis).ti,ab,kw | 238094 |
| 3 | or/1-2 | 262902 |
| 4 | exp exercise/ or endurance/ or exp physical activity/ or training/ or exp kinesiotherapy/ or exp exercise therapy/ or dancing/ or gardening/ or exp sport/ or housekeeping/ or exp ergometer/ | 949105 |
| 5 | (exercis* or physiotherap* or kinesiotherap* or aerobics or athletic* or workout or gym or work* out or (activity adj2 prescri*)).ti,ab,kw | 505548 |
| 6 | (physical* adj3 (activ* or fit* or condition* or program* or train*)).ti,ab,kw | 227438 |
| 7 | (activ* adj3 (motor or living or play* or gaming or game* or lifestyle or behavio?r* or transport* or commut* or transit* or travel* or aerobic*)).ti,ab,kw | 209244 |
| 8 | (crossfit* or HIIT or HIIE or plyometric* or personal train* or bodybuild* or body build* or weightlift* or (weight* adj2 lift*)).ti,ab,kw | 9967 |
| 9 | ((train* or condition* or program* or session*) adj2 (weight* or strength* or enduranc* or resistance or athletic* or fitness or aerobic* or interval* or flexibility or balance or muscle* or muscular or circuit or isometric* or isotonic* or agility or power)).ti,ab,kw | 99502 |
| 10 | (treadmill* or walk* or jog* or swim* or running or runner* or marathon* or triathl* or dance or dancing or climb* or cycling or bicycl* or skateboard* or rowing or rower or skate or skating or ski or skiing or hike or hiking or jump or jumping).ti,ab,kw | 493114 |
| 11 | (tai ji or tai chi or yoga or pilates or stretching or movement or qigong or qi gong or barre or tae bo).ti,ab,kw | 369699 |
| 12 | (gardening or stair* or housework or yardwork* or exergam* or wii fit or zwift or peloton or kinect or ergometer*).ti,ab,kw | 36028 |
| 13 | (martial art* or boxing or kickboxing or karate or judo or jiu jitsu or jiujitsu or taekwondo or tae kwon do or fencing or wrestl* or aikido or fight* or grappl*).ti,ab,kw | 53717 |
| 14 | (sport* or golf* or aquatic* or gymnastic* or tennis or badminton or volleyball or ball or basketball or baseball or bowling or lacrosse or racquet* or softball or polo or cricket or hockey or football or soccer or rugby or handball or mountaineer* or "track and field").ti,ab,kw | 240815 |
| 15 | or/4-14 | 2093939 |
| 16 | 3 and 15 | 5888 |
| 17 | exp clinical trial/ or exp controlled study/ or exp "clinical trial (topic)"/ or cross-sectional study/ or exp case control study/ or exp longitudinal study/ or retrospective study/ or cohort analysis/ or prospective study/ | 10786980 |
| 18 | (case control or case series or cohort* or longitudinal or retrospective or prospective or interrupted time series or cross sectional).tw | 3465025 |
| 19 | ((Follow up or followup or control*) adj2 (study or studies)).tw. | 406241 |
| 20 | (random* or nonrandom* or pseudorandom* or placebo* or groups).ab | 4307767 |
| 21 | trial.ti,ab | 950638 |
| 22 | or/17-21 | 13506852 |
| 23 | 16 and 22 | 2948 |
| 24 | (exp animal/ or nonhuman/) NOT exp human/ | 6637674 |
| 25 | 23 not 24 | 2505 |
| 26 | limit 25 to english language | 2439 |
| 27 | limit 26 to yr="2011 -Current" | 1866 |

CENTRAL SEARCH STRATEGY (10^th^ January 2023)

| 1 | exp inflammatory bowel diseases/ | 3325 |
| --- | --- | --- |
| 2 | (IBD or inflammatory bowel* or crohn* or cleron disease or enteritis or enterocolitis or ileocolitis or ileitis or ileitide* or colitis or proctocolitis or pancolitis or proctosigmoiditis or rectocolitis or rectosigmoiditis or (chronic* adj2 (inflammat* or ulcer*) adj3 (colon* or bowel* or intestinal or intestine?)) or colorectitis or proctitis).ti,ab,kw | 14727 |
| 3 | or/1-2 | 14920 |
| 4 | exp exercise/ or exp exercise movement techniques/ or exp exercise therapy/ or dancing/ or gardening/ or exp sports/ | 39435 |
| 5 | (exercis* or physiotherap* or kinesiotherap* or aerobics or athletic* or workout or gym or work* out or (activity adj2 prescri*)).ti,ab,kw | 190513 |
| 6 | (physical* adj3 (activ* or fit* or condition* or program* or train*)).ti,ab,kw | 46503 |
| 7 | (activ* adj3 (motor or living or play* or gaming or game* or lifestyle or behavio?r* or transport* or commut* or transit* or travel* or aerobic*)).ti,ab,kw | 23128 |
| 8 | (crossfit* or HIIT or HIIE or plyometric* or personal train* or bodybuild* or body build* or weightlift* or (weight* adj2 lift*)).ti,ab,kw | 3090 |
| 9 | ((train* or condition* or program* or session*) adj2 (weight* or strength* or enduranc* or resistance or athletic* or fitness or aerobic* or interval* or flexibility or balance or muscle* or muscular or circuit or isometric* or isotonic* or agility or power)).ti,ab,kw | 38779 |
| 10 | (treadmill* or walk* or jog* or swim* or running or runner* or marathon* or triathl* or dance or dancing or climb* or cycling or bicycl* or skateboard* or rowing or rower or skate or skating or ski or skiing or hike or hiking or jump or jumping).ti,ab,kw | 58071 |
| 11 | (tai ji or tai chi or yoga or pilates or stretching or movement or qigong or qi gong or barre or tae bo).ti,ab,kw | 37372 |
| 12 | (gardening or stair* or housework or yardwork* or exergam* or wii fit or zwift or peloton or kinect or ergometer*).ti,ab,kw | 7860 |
| 13 | (martial art* or boxing or kickboxing or karate or judo or jiu jitsu or jiujitsu or taekwondo or tae kwon do or fencing or wrestl* or aikido or fight* or grappl*).ti,ab,kw | 1905 |
| 14 | (sport* or golf* or aquatic* or gymnastic* or tennis or badminton or volleyball or ball or basketball or baseball or bowling or lacrosse or racquet* or softball or polo or cricket or hockey or football or soccer or rugby or handball or mountaineer* or "track and field").ti,ab,kw | 16137 |
| 15 | or/4-14 | 281724 |
| 16 | 3 and 15 | 863 |
| 17 | limit 16 to english language | 509 |
| 18 | limit 17 to yr="2011 -Current" | 405 |

PSYCINFO SEARCH STRATEGY (10^th^ January 2023)

| 1 | exp colon disorders/ | 4467 |
| --- | --- | --- |
| 2 | (IBD or inflammatory bowel* or crohn* or cleron disease or enteritis or enterocolitis or ileocolitis or ileitis or ileitide* or colitis or proctocolitis or pancolitis or proctosigmoiditis or rectocolitis or rectosigmoiditis or (chronic* adj2 (inflammat* or ulcer*) adj3 (colon* or bowel* or intestinal or intestine?)) or colorectitis or proctitis).ti,ab,id | 2286 |
| 3 | or/1-2 | 5629 |
| 4 | exp physical activity/ or physical fitness/ or active living/ or movement therapy/ or physical therapy/ or dance/ or horticulture therapy/ or athletic participation/ or exp sports/ or athletic performance/ or athletic training/ or dance therapy/ | 90301 |
| 5 | (exercis* or physiotherap* or kinesiotherap* or aerobics or athletic* or workout or gym or work* out or (activity adj2 prescri*)).ti,ab,id | 88811 |
| 6 | (physical* adj3 (activ* or fit* or condition* or program* or train*)).ti,ab,id | 52338 |
| 7 | (activ* adj3 (motor or living or play* or gaming or game* or lifestyle or behavio?r* or transport* or commut* or transit* or travel* or aerobic*)).ti,ab,id | 56984 |
| 8 | (crossfit* or HIIT or HIIE or plyometric* or personal train* or bodybuild* or body build* or weightlift* or (weight* adj2 lift*)).ti,ab,id | 1904 |
| 9 | ((train* or condition* or program* or session*) adj2 (weight* or strength* or enduranc* or resistance or athletic* or fitness or aerobic* or interval* or flexibility or balance or muscle* or muscular or circuit or isometric* or isotonic* or agility or power)).ti,ab,id | 14568 |
| 10 | (treadmill* or walk* or jog* or swim* or running or runner* or marathon* or triathl* or dance or dancing or climb* or cycling or bicycl* or skateboard* or rowing or rower or skate or skating or ski or skiing or hike or hiking or jump or jumping).ti,ab,id | 80345 |
| 11 | (tai ji or tai chi or yoga or pilates or stretching or movement or qigong or qi gong or barre or tae bo).ti,ab,id | 110520 |
| 12 | (gardening or stair* or housework or yardwork* or exergam* or wii fit or zwift or peloton or kinect or ergometer*).ti,ab,id | 5753 |
| 13 | (martial art* or boxing or kickboxing or karate or judo or jiu jitsu or jiujitsu or taekwondo or tae kwon do or fencing or wrestl* or aikido or fight* or grappl*).ti,ab,id | 21941 |
| 14 | (sport* or golf* or aquatic* or gymnastic* or tennis or badminton or volleyball or ball or basketball or baseball or bowling or lacrosse or racquet* or softball or polo or cricket or hockey or football or soccer or rugby or handball or mountaineer* or "track and field").ti,ab,id | 53083 |
| 15 | or/4-14 | 407851 |
| 16 | 3 and 15 | 376 |
| 17 | exp clinical trials/ or cohort analysis/ or followup studies/ or exp longitudinal studies/ or retrospective studies/ | 43990 |
| 18 | ("0300" or "0430" or "0450" or "0451" or "0453").md | 251936 |
| 19 | (random* or placebo* or groups).ab | 697945 |
| 20 | trial.ti,ab | 113434 |
| 21 | (case control or case series or cohort* or longitudinal or retrospective or prospective or interrupted time series or cross sectional).tw | 348518 |
| 22 | ((Follow up or followup or control*) adj2 (study or studies)).tw. | 48185 |
| 23 | or/17-22 | 1120590 |
| 24 | 16 and 23 | 158 |
| 25 | ("20" not "10").po | 371132 |
| 26 | 24 not 25 | 154 |
| 27 | limit 26 to english language | 152 |
| 28 | limit 27 to yr="2011 -Current" | 82 |

CINAHL SEARCH STRATEGY (10^th^ January 2023)

| 1 | (MH "inflammatory bowel diseases+") | 16636 |
| --- | --- | --- |
| 2 | AB(IBD OR (inflammatory N1 bowel*) OR crohn* OR "cleron disease" OR enteritis OR enterocolitis OR ileocolitis OR ileitis OR ileitide* OR colitis OR proctocolitis OR pancolitis OR proctosigmoiditis OR rectocolitis OR rectosigmoiditis OR (chronic* N2 (inflammat* OR ulcer*) N3 (colon* OR bowel* OR intestinal OR intestine OR intestines)) OR colorectitis OR proctitis) | 18034 |
| 3 | S1 OR S2 | 25544 |
| 4 | (MH exercise+) OR (MH "physical activity") OR (MH "physical fitness+") OR (MH "therapeutic exercise+") OR (MH dancing+) OR (MH horticulture) OR (MH sports+) | 253806 |
| 5 | AB(exercis* OR physiotherap* OR kinesiotherap* OR aerobics OR athletic* OR workout OR gym OR work* out OR (activity N2 prescri*)) | 138471 |
| 6 | AB(physical* N3 (activ* OR fit* OR condition* OR program* OR train*)) | 73731 |
| 7 | AB(activ* N3 (motor OR living OR play* OR gaming OR game* OR lifestyle OR behavior* OR behaviour* OR transport* OR commut* OR transit* OR travel* OR aerobic*)) | 41137 |
| 8 | AB(crossfit* OR HIIT OR HIIE OR plyometric* OR (personal N1 train*) OR bodybuild* OR (body N1 build*) OR weightlift* OR (weight* N2 lift*)) | 4017 |
| 9 | AB((train* OR condition* OR program* OR session*) N2 (weight* OR strength* OR enduranc* OR resistance OR athletic* OR fitness OR aerobic* OR interval* OR flexibility OR balance OR muscle* OR muscular OR circuit OR isometric* OR isotonic* OR agility OR power)) | 32023 |
| 10 | AB(treadmill* OR walk* OR jog* OR swim* OR running OR runner* OR marathon* OR triathl* OR dance OR dancing OR climb* OR cycling OR bicycl* OR skateboard* OR rowing OR rower OR skate OR skating OR ski OR skiing OR hike OR hiking OR jump OR jumping) | 86636 |
| 11 | AB("tai ji" OR "tai chi" OR yoga OR pilates OR stretching OR movement OR qigong OR "qi gong" OR barre OR "tae bo") | 72219 |
| 12 | AB(gardening OR stair* OR housework OR yardwork* OR exergam* OR "wii fit" OR zwift OR peloton OR kinect OR ergometer*) | 8724 |
| 13 | AB((martial N1 art*) OR boxing OR kickboxing OR karate OR judo OR "jiu jitsu" OR jiujitsu OR taekwondo OR "tae kwon do" OR fencing OR wrestl* OR aikido OR fight* OR grappl*) | 10977 |
| 14 | AB(sport* OR golf* OR aquatic* OR gymnastic* OR tennis OR badminton OR volleyball OR ball OR basketball OR baseball OR bowling OR lacrosse OR racquet* OR softball OR polo OR cricket OR hockey OR football OR soccer OR rugby OR handball OR mountaineer* OR "track and field") | 56178 |
| 15 | S4 OR S5 OR S6 OR S7 OR S8 OR S9 OR S10 OR S11 OR S12 OR S13 OR S14 | 495385 |
| 16 | S3 AND S15 | 594 |
| 17 | (ZT "randomized controlled trial") or (ZT "clinical trial") | 229663 |
| 18 | (MH "experimental studies+") or (MH "retrospective design") or (MH "cross sectional studies") or (MH "case control studies+") or (MH "prospective studies+") | 1236145 |
| 19 | AB(random* OR placebo* OR groups OR trial) | 1074661 |
| 20 | TI(trial) | 153426 |
| 21 | ("case control" or "case series" or cohort* or longitudinal or retrospective or prospective or "interrupted time series" or "cross sectional") | 1181394 |
| 22 | ((Follow up or followup or control*) N2 (study or studies)) | 158678 |
| 23 | S17 OR S18 OR S19 OR S20 OR S21 OR S22 | 2053993 |
| 24 | S16 AND S23 | 260 |
| 25 | (MH vertebrates+) NOT (MH human) | 202811 |
| 26 | S24 NOT S25 | 251 |
| 27 | S26 Limiters - English language | 249 |
| 28 | S27 Limiters - Published Date: 20110101- | 185 |

SCOPUS SEARCH STRATEGY (10^th^ January 2023)

| 1 | TITLE-ABS-KEY(IBD OR (inflammatory W/1 bowel*) OR crohn* OR "cleron disease" OR enteritis OR enterocolitis OR ileocolitis OR ileitis OR ileitide* OR colitis OR proctocolitis OR pancolitis OR proctosigmoiditis OR rectocolitis OR rectosigmoiditis OR (chronic* W/2 (inflammat* OR ulcer*) W/3 (colon* OR bowel* OR intestinal OR intestine OR intestines)) OR colorectitis OR proctitis) | 253551 |
| --- | --- | --- |
| 2 | TITLE-ABS-KEY(exercis* OR physiotherap* OR kinesiotherap* OR aerobics OR athletic* OR workout OR gym OR work* out OR (activity W/2 prescri*)) | 802174 |
| 3 | TITLE-ABS-KEY(physical* W/3 (activ* OR fit* OR condition* OR program* OR train*)) | 371862 |
| 4 | TITLE-ABS-KEY(activ* W/3 (motor OR living OR play* OR gaming OR game* OR lifestyle OR behavior* OR behaviour OR transport* OR commut* OR transit* OR travel* OR aerobic*)) | 475085 |
| 5 | TITLE-ABS-KEY(crossfit* OR HIIT OR HIIE OR plyometric* OR (personal W/1 train*) OR bodybuild* OR (body W/1 build*) OR weightlift* OR (weight* W/2 lift*)) | 25224 |
| 6 | TITLE-ABS-KEY((train* OR condition* OR program* OR session*) W/2 (weight* OR strength* OR enduranc* OR resistance OR athletic* OR fitness OR aerobic* OR interval* OR flexibility OR balance OR muscle* OR muscular OR circuit OR isometric* OR isotonic* OR agility OR power)) | 256446 |
| 7 | TITLE-ABS-KEY(treadmill* OR walk* OR jog* OR swim* OR running OR runner* OR marathon* OR triathl* OR dance OR dancing OR climb* OR cycling OR bicycl* OR skateboard* OR rowing OR rower OR skate OR skating OR ski OR skiing OR hike OR hiking OR jump OR jumping) | 1306858 |
| 8 | TITLE-ABS-KEY("tai ji" OR "tai chi" OR yoga OR pilates OR stretching OR movement OR qigong OR "qi gong" OR barre OR "tae bo") | 1401895 |
| 9 | TITLE-ABS-KEY(gardening OR stair* OR housework OR yardwork* OR exergam* OR "wii fit" OR zwift OR peloton OR kinect OR ergometer*) | 70585 |
| 10 | TITLE-ABS-KEY((martial W/1 art*) OR boxing OR kickboxing OR karate OR judo OR "jiu jitsu" OR jiujitsu OR taekwondo OR "tae kwon do" OR fencing OR wrestl* OR aikido OR fight* OR grappl*) | 181920 |
| 11 | TITLE-ABS-KEY(sport* OR golf* OR aquatic* OR gymnastic* OR tennis OR badminton OR volleyball OR ball OR basketball OR baseball OR bowling OR lacrosse OR racquet* OR softball OR polo OR cricket OR hockey OR football OR soccer OR rugby OR handball OR mountaineer* OR "track and field") | 705573 |
| 12 | #2 OR #3 OR #4 OR #5 OR #6 OR #7 OR #8 | 4229318 |
| 13 | #9 OR #10 OR #11 | 942172 |
| 14 | #12 OR #13 | 4964229 |
| 15 | #1 AND #14 | 7172 |
| 16 | INDEXTERMS("randomized controlled trial" OR "controlled clinical trial" OR "clinical trials as topic" OR "randomized controlled trials as topic" or "non-randomized controlled trials as topic" or "clinical trial" or "epidemiologic studies" or "case-control studies" or "retrospective studies" or "cohort studies" or "follow-up studies" or "longitudinal studies" or "prospective studies" or "controlled before-after studies" or "cross-sectional studies" or "historically controlled study" or "interrupted time series analysis") | 4220713 |
| 17 | ABS(random* OR placebo* OR groups) | 9128650 |
| 18 | TITLE-ABS(trial) | 1489880 |
| 19 | TITLE-ABS-KEY("case control" or "case series" or cohort* or longitudinal or retrospective or prospective or "interrupted time series" or "cross sectional") | 4047737 |
| 20 | TITLE-ABS-KEY(("Follow up" or followup or control*) W/2 (study or studies)) | 7397398 |
| 21 | #16 OR #17 OR #18 OR #19 OR #20 | 17141704 |
| 22 | #15 AND #21 | 3918 |
| 23 | INDEXTERMS(animal*) AND NOT INDEXTERMS(human*) | 5116942 |
| 24 | #22 AND NOT #23 | 3329 |
| 25 | #24 AND ( LIMIT-TO ( LANGUAGE , "English" ) ) | 3150 |
| 26 | #25 AND PUBYEAR AFT 2010 | 1902 |

WEB OF SCIENCE CORE COLLECTION SEARCH STRATEGY (10^th^ January 2023)

| 1 | TS=(IBD OR (inflammatory NEAR/1 bowel*) OR crohn* OR "cleron disease" OR enteritis OR enterocolitis OR ileocolitis OR ileitis OR ileitide* OR colitis OR proctocolitis OR pancolitis OR proctosigmoiditis OR rectocolitis OR rectosigmoiditis OR (chronic* NEAR/2 (inflammat* OR ulcer*) NEAR/3 (colon* OR bowel* OR intestinal OR intestine OR intestines)) OR colorectitis OR proctitis) | 227795 |
| --- | --- | --- |
| 2 | TS=(exercis* OR physiotherap* OR kinesiotherap* OR aerobics OR athletic* OR workout OR gym OR work* out OR (activity NEAR/2 prescri*)) | 1100265 |
| 3 | TS=(physical* NEAR/3 (activ* OR fit* OR condition* OR program* OR train*)) | 302204 |
| 4 | TS=(activ* NEAR/3 (motor OR living OR play* OR gaming OR game* OR lifestyle OR behavior* OR behaviour OR transport* OR commut* OR transit* OR travel* OR aerobic*)) | 266662 |
| 5 | TS=(crossfit* OR HIIT OR HIIE OR plyometric* OR (personal NEAR/1 train*) OR bodybuild* OR (body NEAR/1 build*) OR weightlift* OR (weight* NEAR/2 lift*)) | 13657 |
| 6 | TS=((train* OR condition* OR program* OR session*) NEAR/2 (weight* OR strength* OR enduranc* OR resistance OR athletic* OR fitness OR aerobic* OR interval* OR flexibility OR balance OR muscle* OR muscular OR circuit OR isometric* OR isotonic* OR agility OR power)) | 183117 |
| 7 | TS=(treadmill* OR walk* OR jog* OR swim* OR running OR runner* OR marathon* OR triathl* OR dance OR dancing OR climb* OR cycling OR bicycl* OR skateboard* OR rowing OR rower OR skate OR skating OR ski OR skiing OR hike OR hiking OR jump OR jumping) | 2826670 |
| 8 | TS=("tai ji" OR "tai chi" OR yoga OR pilates OR stretching OR movement OR qigong OR "qi gong" OR barre OR "tae bo") | 1021394 |
| 9 | TS=(gardening OR stair* OR housework OR yardwork* OR exergam* OR "wii fit" OR zwift OR peloton OR kinect OR ergometer*) | 104333 |
| 10 | TS=((martial NEAR/1 art*) OR boxing OR kickboxing OR karate OR judo OR "jiu jitsu" OR jiujitsu OR taekwondo OR "tae kwon do" OR fencing OR wrestl* OR aikido OR fight* OR grappl*) | 357159 |
| 11 | TS=(sport* OR golf* OR aquatic* OR gymnastic* OR tennis OR badminton OR volleyball OR ball OR basketball OR baseball OR bowling OR lacrosse OR racquet* OR softball OR polo OR cricket OR hockey OR football OR soccer OR rugby OR handball OR mountaineer* OR "track and field") | 563732 |
| 12 | #2 OR #3 OR #4 OR #5 OR #6 OR #7 OR #8 OR #9 OR #10 OR #11 | 5927081 |
| 13 | #1 AND #12 | 7359 |
| 14 | TS=((controlled NEAR/1 trial*) or (clinical NEAR/1 trial*) or "case control" or "case series" or cohort* or longitudinal or retrospective or prospective or "interrupted time series" or "cross sectional") | 3441430 |
| 15 | TS=(("Follow up" or followup or control*) NEAR/2 (study or studies)) | 425802 |
| 16 | AB=(random* OR placebo* OR groups OR trial) | 6736740 |
| 17 | TI=(trial) | 471908 |
| 18 | #14 OR #15 OR #16 OR #17 | 9053416 |
| 19 | #13 AND #18 | 2589 |
| 20 | TS=(animal*) NOT TS=(human*) | 948668 |
| 21 | #19 NOT #20 | 2421 |
| 22 | #21 AND Language: (English) | 2325 |
| 23 | #22 \| IC Timespan=2011-2021 | 1544 |

PEDRO SEARCH STRATEGY (10^th^ January 2023)

| 1 | Abstract & Title: "inflammatory bowel" \| Method: clinical trial \| Published since: 2011 | 5 |
| --- | --- | --- |
| 2 | Abstract & Title: IBD \| Method: clinical trial \| Published since: 2011 | 3 |
| 3 | Abstract & Title: crohn* \| Method: clinical trial \| Published since: 2011 | 7 |
| 4 | Abstract & Title: colitis \| Method: clinical trial \| Published since: 2011 | 0 |
| 5 | Total unique | 10 |

PRISMA CHECKLIST

| **Section and Topic** | **Item #** |  | | **Checklist item** | **Location where item is reported** |
| --- | --- | --- | --- | --- | --- |
|  | | | **TITLE** | |  |
| Title | 1 |  | | Identify the report as a systematic review. | 1 |
|  | | | **ABSTRACT** | |  |
| Abstract | 2 |  | | See the PRISMA 2020 for Abstracts checklist. | 2 |
|  | | | **INTRODUCTION** | |  |
| Rationale | 3 |  | | Describe the rationale for the review in the context of existing knowledge. | 4, 5 |
| Objectives | 4 |  | | Provide an explicit statement of the objective(s) or question(s) the review addresses. | 5 |
|  | | | **METHODS** | |  |
| Eligibility criteria | 5 |  | | Specify the inclusion and exclusion criteria for the review and how studies were grouped for the syntheses. | 6 |
| Information sources | 6 |  | | Specify all databases, registers, websites, organisations, reference lists and other sources searched or consulted to identify studies. Specify the date when each source was last searched or consulted. | 6, 7 |
| Search strategy | 7 |  | | Present the full search strategies for all databases, registers and websites, including any filters and limits used. | 6, 7 |
| Selection process | 8 |  | | Specify the methods used to decide whether a study met the inclusion criteria of the review, including how many reviewers screened each record and each report retrieved, whether they worked independently, and if applicable, details of automation tools used in the process. | 7 |
| Data collection process | 9 |  | | Specify the methods used to collect data from reports, including how many reviewers collected data from each report, whether they worked independently, any processes for obtaining or confirming data from study investigators, and if applicable, details of automation tools used in the process. | 7 |
| Data items | 10a |  | | List and define all outcomes for which data were sought. Specify whether all results that were compatible with each outcome domain in each study were sought (e.g. for all measures, time points, analyses), and if not, the methods used to decide which results to collect. | 6 |
|  | 10b |  | | List and define all other variables for which data were sought (e.g. participant and intervention characteristics, funding sources). Describe any assumptions made about any missing or unclear information. | 6, 7 |
| Study risk of bias assessment | 11 |  | | Specify the methods used to assess risk of bias in the included studies, including details of the tool(s) used, how many reviewers assessed each study and whether they worked independently, and if applicable, details of automation tools used in the process. | 8 |
| Effect measures | 12 |  | | Specify for each outcome the effect measure(s) (e.g. risk ratio, mean difference) used in the synthesis or presentation of results. | 8 |
| Synthesis methods | 13a |  | | Describe the processes used to decide which studies were eligible for each synthesis (e.g. tabulating the study intervention characteristics and comparing against the planned groups for each synthesis (item #5)). | 8 |
|  | 13b |  | | Describe any methods required to prepare the data for presentation or synthesis, such as handling of missing summary statistics, or data conversions. | 8 |
|  | 13c |  | | Describe any methods used to tabulate or visually display results of individual studies and syntheses. | 7, 8 |
|  | 13d |  | | Describe any methods used to synthesize results and provide a rationale for the choice(s). If meta-analysis was performed, describe the model(s), method(s) to identify the presence and extent of statistical heterogeneity, and software package(s) used. | 8 |
|  | 13e |  | | Describe any methods used to explore possible causes of heterogeneity among study results (e.g. subgroup analysis, meta-regression). | 8 |
|  | 13f |  | | Describe any sensitivity analyses conducted to assess robustness of the synthesized results. | 8 |
| Reporting bias assessment | 14 |  | | Describe any methods used to assess risk of bias due to missing results in a synthesis (arising from reporting biases). | 8 |
| Certainty assessment | 15 |  | | Describe any methods used to assess certainty (or confidence) in the body of evidence for an outcome. | 9 |
|  | | | **RESULTS** | |  |
| Study selection | 16a |  | | Describe the results of the search and selection process, from the number of records identified in the search to the number of studies included in the review, ideally using a flow diagram. | 10 |
|  | 16b |  | | Cite studies that might appear to meet the inclusion criteria, but which were excluded, and explain why they were excluded. | 27 |
| Study characteristics | 17 |  | | Cite each included study and present its characteristics. | 23 |
| Risk of bias in studies | 18 |  | | Present assessments of risk of bias for each included study. | 10, 28 |
| Results of individual studies | 19 |  | | For all outcomes, present, for each study: (a) summary statistics for each group (where appropriate) and (b) an effect estimate and its precision (e.g. confidence/credible interval), ideally using structured tables or plots. | 10-14 |
| Results of syntheses | 20a |  | | For each synthesis, briefly summarise the characteristics and risk of bias among contributing studies. | 10 |
|  | 20b |  | | Present results of all statistical syntheses conducted. If meta-analysis was done, present for each the summary estimate and its precision (e.g. confidence/credible interval) and measures of statistical heterogeneity. If comparing groups, describe the direction of the effect. | 10-14 |
|  | 20c |  | | Present results of all investigations of possible causes of heterogeneity among study results. | 11 |
|  | 20d |  | | Present results of all sensitivity analyses conducted to assess the robustness of the synthesized results. | 11 |
| Reporting biases | 21 |  | | Present assessments of risk of bias due to missing results (arising from reporting biases) for each synthesis assessed. | 10 |
| Certainty of evidence | 22 |  | | Present assessments of certainty (or confidence) in the body of evidence for each outcome assessed. | 26 |
|  | | | **DISCUSSION** | |  |
| Discussion | 23a |  | | Provide a general interpretation of the results in the context of other evidence. | 15 |
|  | 23b |  | | Discuss any limitations of the evidence included in the review. | 16 |
|  | 23c |  | | Discuss any limitations of the review processes used. | 16 |
|  | 23d |  | | Discuss implications of the results for practice, policy, and future research. | 17 |
|  | | | **OTHER INFORMATION** | |  |
| Registration and protocol | 24a |  | | Provide registration information for the review, including register name and registration number, or state that the review was not registered. | 6 |
|  | 24b |  | | Indicate where the review protocol can be accessed, or state that a protocol was not prepared. | 6 |
|  | 24c |  | | Describe and explain any amendments to information provided at registration or in the protocol. |  |
| Support | 25 |  | | Describe sources of financial or non-financial support for the review, and the role of the funders or sponsors in the review. | 18 |
| Competing interests | 26 |  | | Declare any competing interests of review authors. | 18 |
| Availability of data, code and other materials | 27 |  | | Report which of the following are publicly available and where they can be found: template data collection forms; data extracted from included studies; data used for all analyses; analytic code; any other materials used in the review. | 19 |
